# Experiences participating in federal nutrition assistance programs during the early months of the COVID-19 pandemic: an investigation in Vermont

**DOI:** 10.1186/s12937-024-00963-z

**Published:** 2024-07-15

**Authors:** Emma H. Spence, Meredith T. Niles, Farryl Bertmann, Emily H. Belarmino

**Affiliations:** 1 Food Systems Program, University of Vermont, 109 Carrigan Drive, Burlington, VT 05405 USA; 2 Department of Nutrition and Food Sciences, University of Vermont, 109 Carrigan Drive, Burlington, VT 05405 USA; 3 Gund Institute for Environment, University of Vermont, 210 Colchester Ave, Burlington, VT 05405 USA

**Keywords:** Food security, Stress, Fruit intake, Vegetable intake, Nutrition assistance, SNAP, WIC, School meals

## Abstract

**Background:**

Federal nutrition assistance programs serve as safety nets for many American households, and participation has been linked to increased food security and, in some instances, improved diet quality and mental health outcomes. The COVID-19 pandemic brought new and increased economic, social, and psychological challenges, necessitating inquiry into how nutrition assistance programs are functioning and associated with public health outcomes.

**Methods:**

Using data from a representative statewide survey administered in Vermont (*n* = 600) between July and September 2020, we examined participant experiences with major federal nutrition assistance programs: the Supplemental Nutrition Assistance Program (SNAP), the Special Supplemental Nutrition Program for Women, Infants, and Children (WIC), and school meal programs. We explored quantitative and qualitative responses regarding perceptions of program utility, and used nearest neighbors matching analyses in combination with bivariate statistical tests to assess associations between program participation and food insecurity, perceived stress, and fruit and vegetable intake as indicators of dietary quality.

**Results:**

One in four respondents (27.3%) used at least one federal nutrition assistance program. As compared to non-participants, we found higher rates of food insecurity among program participants (57.5% vs. 18.1%; *p* < 0.001), an association that persisted even when we compared similar households using matching techniques (*p* ≤ 0.001). From matched analyses, we found that, compared to low-income non-participants, low-income program participants were less likely to meet fruit intake recommendations (*p* = 0.048) and that low-income SNAP and WIC participants were less likely to meet vegetable intake recommendations (*p* = 0.035). We also found lower rates of perceived stress among low-income school meal participant households compared to low-income non-participants (*p* = 0.039). Despite these mixed outcomes, participants broadly valued federal nutrition assistance programs, characterizing them as helpful or easy to use.

**Conclusions:**

We found that federal nutrition assistance programs as a group were not sufficient to address food insecurity and stress or increase fruit and vegetable intake in the state of Vermont during the early months of the COVID-19 pandemic. Nonetheless, participants perceived benefits from participation in these programs. Optimizing the utility of nutrition assistance programs depends on critical examination of their functioning under conditions of great stress.

**Supplementary Information:**

The online version contains supplementary material available at 10.1186/s12937-024-00963-z.

## Background

Collectively, federal food and nutrition assistance programs in the United States impact tens of millions of Americans annually [1]. The largest domestic programs include the Supplementary Nutrition Assistance Program (SNAP), the Special Supplemental Nutrition Program for Women, Infants, and Children (WIC), the National School Lunch Program (NSLP), and the School Breakfast Program (SBP). Collectively, these programs have been credited as essential safety nets in ensuring adequate nutrition for many who live at the margins of hunger and food insecurity. The COVID-19 pandemic and the resulting economic recession led the Federal Government to expand these programs and adjust their operations to protect the health and safety of both participants and staff. As economic recovery continues, there is a need for research on the impacts of the pandemic on food and nutrition security and evaluation of the role of food and nutrition assistance programs in supporting vulnerable Americans.

The Supplemental Nutrition Assistance Program (SNAP) provides low-income households with benefits to buy food, with the benefit amount varying based on a household’s income and certain expenses. In 2020, SNAP reached 41.2 million people or about 12% of the US population. Most who meet the program’s income guidelines are eligible to participate. In nationally representative studies, SNAP participants are more likely to be female [2], and tend to be younger than eligible non-participants [3]. As of 2018, a significant majority (81%) of SNAP households contained at least one child, elderly individual, or individual with a disability, and 61% of those with children were single-adult households [2]. Additionally, 81% of SNAP households had no cash income of any kind [2]. Monthly SNAP participation has fluctuated significantly since the program’s origin, peaking at 15.2% of residents nationwide in 2013 after the Great Recession and subsequently declining [4]. Notably, early findings suggest that nationally, among households with children, participation in SNAP declined in the early months of the COVID-19 pandemic [5]. Program administration challenges, including failures in customer service and difficulties navigating administrative bureaucracy are common complaints, specifically during the application and renewal processes [6]. Some SNAP participants have expressed concerns over benefit adequacy [6–8]; however, research has also found SNAP participants to express the belief that the program fulfilled its essential function of providing enough supplemental food to make ends meet, that the electronic benefits transfer (EBT) card format was easy to use, and that benefits were dependable [6].

WIC provides supplemental foods and other support to pregnant and postpartum women and infants and children up to age five who are at nutritional risk and living in low-income households. In 2020, WIC served approximately seven million Americans [9], including roughly half of all babies born in the US [10]. In contrast to fluctuations in SNAP participation, WIC enrollment has been relatively stable, with rates roughly reflecting broader sociodemographic trends [11]. However, there is significant variability in the duration of WIC enrollment, with characteristics such as lower household income increasing the probability of sustained enrollment [12] and factors such as breastfeeding and home ownership associated with lower intent to maintain enrollment [13]. Overall estimates of WIC-eligible non-participation rates range as high as 50% [14]. In a population-based randomized survey of 1,634 postpartum women in New York City, WIC-eligible mothers facing structural barriers such as lack of transportation, unplanned pregnancy, and limited social supports were less likely to participate in WIC [15]. Multiple other factors have been found to influence participants’ views of the program including logistical challenges in meeting time and transportation demands; program administration challenges, including failures in communication and organization; and challenges in the retail environment, including inconsistency and/or difficulty identifying eligible foods, lack of choice, lack of training of store employees, and perceptions of stigma [7, 14, 16–19]. In a mixed methods study of 150 WIC caregivers in Illinois, participants also assessed the value of the program against the time and effort required to maintain eligibility [17]. During pregnancy and infancy, most participants believed benefits to be worth the time and effort (70%; 91%), hypothesized to be a function of the high cost of formula. However, only 36% believed the child program package value to be worth the effort once past reliance on formula. Additionally, the more restrictive selection options under WIC may make WIC more difficult and stigmatizing to use than SNAP but are viewed by some as a useful incentive to eat more healthfully [16, 17].

The National School Lunch Program (NSLP) and the School Breakfast Program (SBP) – sometimes referred to collectively as school meal programs – provide nutritious meals at low or no cost to children in participating schools. Prior to the pandemic, nearly 30 million schoolchildren received lunch through the NSLP each day and nearly 15 million received breakfast through the SBP [20]. Under standard operation of the NSLP and SBP, school meals are available for purchase, and some students receive meals for free or at a reduced price, funded by the federal government. Universal school meals is a variation of the program in which meals are made available to all students at no cost. School meal participation is highest among children eligible for free meals, and especially where universal free meals are offered [21, 22]. Multiple studies suggest that parental and student perceptions of school meals’ healthfulness are significantly associated with program participation [23–25]. Sociocultural preferences and logistical challenges have also been shown to affect school meal participation. Examples include valuing of family mealtime, as well as concerns over school meal quality and food choice [26–28]. When the pandemic disrupted in-person education and meal services, the US Department of Agriculture created the Pandemic Electronic Benefit Transfer (P-EBT) program, which reimbursed eligible households for the value of school meals their children missed because of the pandemic, and supported new meal service locations and methods to improve access and safety [29].

### Federal nutrition assistance programs, food security, diet quality and mental health

It is difficult to assess a causal relationship between federal food and nutrition assistance programs and food security status, largely since households experiencing food insecurity are significantly more likely to participate in such programs. For example, in 2019, roughly 58% of food-insecure households participated in SNAP, WIC, and/or the NSLP [30]. However, studies attempting to control for selection bias suggest that SNAP participation may reduce the prevalence of food insecurity by as much as 30%, although specific estimates vary [31–34]. In a survey of 9,811 households, Mabli et al. [33] found that SNAP participation among those enrolled for six months was associated with a 7% reduction in household food insecurity as compared to new enrollees, and a 16% reduction for those same new enrollees evaluated again after 6 months. Similarly, reductions in very low food security were 14% and 18%, respectively. Greater benefit allotments were associated with more significant improvements in food security status [33]. However, Leung et al. [35] found no significant improvements in household food security over a three-month period in a sample of newly enrolled SNAP participants, suggesting that duration of enrollment may be relevant. Likewise, studies have found the probability of being food insecure to increase in the last several days of the benefit cycle [36] and if benefits are temporarily lost due to administrative issues with recertification (a phenomenon known as churning) [37].

Both WIC and school meal program participation have been associated with significant food security benefits for children [38–42]. Among WIC households, food insecurity appears to be mediated by earlier program entry and longer duration of participation [39]. Children from food insecure households obtain a larger proportion of their total daily calories and nutrients from school meals as compared to children from highly food secure households [40]. For children who consume both school breakfast and lunch, the two meals have been found to provide nearly half of daily energy intake [41]. Based on higher reported rates of food insecurity during the summer months among households with children receiving free and reduced-price lunch, Huang and Barnidge [44] suggest that National School Lunch Program participation may be associated with a reduction in food insecurity of roughly 14% [42].

The findings of studies into associations between federal nutrition assistance programs and dietary quality may depend on the program. In a systematic review of 25 studies examining diet, Andreyeva, Tripp and Schwartz [43] found that overall caloric, macro and micronutrient intakes were not significantly different between SNAP participants and income eligible non-participants. Results of specific studies on dietary quality are mixed, with some finding that SNAP participants had poorer overall diet quality than both income-eligible and higher income non-participants [43–46], while others have found improvements in dietary quality among SNAP eligible respondents who used the program [47]. Focusing on fruit and vegetable intake, Saxe-Custack et al. [48] recently found that although SNAP participation did not increase the probability of participants meeting national dietary recommendations, it did significantly increase the mean daily consumption of both fruits and vegetables for a cohort of child participants. Others have shown that trends in fruit and vegetable purchasing among SNAP households vary significantly according to the benefit cycle, although they are similar on average to non-participant households [49]. Evidence suggests that specific incentive programs for SNAP participants, including Double-Up Food Bucks and other targeted fruit and vegetable purchasing incentives, may increase intake more than SNAP alone [50, 51].

WIC participation is significantly associated with improved diet quality in children [52, 53]. In an analysis of 1250 children enrolled in WIC, Weinfield et al. [53] also found that longer duration of program participation was associated with significantly higher Healthy Eating Index (HEI) scores as compared to eligible candidates who discontinued participation after infancy. A systematic review by Zhang et al. [54] also shows consistent, although not universal, positive correlations between fruit and vegetable purchasing and/or consumption by WIC participants since WIC food packages were updated in 2009 to include more fruits, vegetables, whole grains, and low-fat dairy, Participation in daily school breakfast and lunch was associated with modestly healthier dietary intakes among 5,106 US school children, ages 4–15 [55]. In a study of 3944 fourth and fifth graders, school lunch eaters had higher average HEI scores than those who ate lunch brought from home (mean HEI: 49.0 vs. 46.1); however, there was no difference in overall HEI score between children who ate breakfast obtained from school and those who obtained their meals from home or a combination of school and home [56].

Food insecurity has been associated with multiple indicators of poor mental health [57–59]. In a systematic review of 12 studies, Bruening et al. [57] identified a bidirectional relationship between food insecurity and negative emotional health in US-based populations. Myers et al. [59] likewise reported significant positive associations between food insecurity and multiple measures of psychological distress based on an assortment of cross-sectional, longitudinal and secondary data studies in numerous countries. Focusing on high-income countries, Maynard et al. [58] found associations between food insecurity and mental health metrics, including symptoms of depression, anxiety and stress, among women in a review of 39 studies. Even more recently, using cross-sectional data from the 2020 U.S. Census Household Pulse Survey (*N* = 63,674), Nagata et al. [60] reported independent associations between food insufficiency and all measured indicators of poor mental health, controlling for sociodemographic covariates. Interestingly, they found that this association was mitigated by receipt of free groceries and meals [60].

Several studies have attempted to examine how nutrition assistance programs may mediate relationships between food insecurity and various measures of mental health. Pulling data from the 2011-12 longitudinal SNAP Food Security Survey, Oddo and Mabli [61] found that, among 3,146 U.S. households, 7.9% fewer household heads reported symptoms of psychological distress after 6 months of SNAP participation, and adjusted models show an associated decrease in psychological distress. Leung et al. [62] also examined the association between food insecurity and depression, as evaluated in the 2005–2010 NHANES dataset, restricted to adults earning no more than 130% of the Federal Poverty Level (FPL). Controlling for sociodemographic and health covariates, they found a significant positive association between food insecurity and depression, but SNAP participation lessened the strength of this relationship. This was also identified during the COVID-19 pandemic. An analysis of 1,256 adults in Massachusetts with incomes ≤ 300% FPL in the first year of the pandemic found those experiencing food insecurity consumed less food compared to those not experiencing food insecurity, but participation in SNAP attenuated this association for highly nutritious foods [63]. However, Adynski et al. [64] found that, controlling for demographic variables, SNAP and WIC participation did not reduce the risk of depressive symptoms in a nationally representative sample from the NHANES 2013–2014 and 2015–2016 cohorts, while elevated levels of food insecurity were associated with higher risks of depressive symptoms.

Responses to the COVID-19 pandemic varied across the country. The predominantly rural state of Vermont was characterized by a relatively robust policy and social response [65]. Rural populations experience higher rates of food insecurity and are more likely participate in nutrition assistance programs as compared to their urban counterparts [30, 66]. Vermont readily accepted and implemented numerous federal waivers made available in association with the Families First Coronavirus Response Act to increase the flexibility of programs in response to COVID-19, from provision of P-EBT to suspension of certain face-to-face interview requirements to the temporary restructuring of school meal delivery [67]. The state also boasted among the lowest COVID-19 caseloads at the time of survey administration [65].

Due to the urgent and persistent nature of the COVID-19 pandemic, there is a continuing need for research on the broader impacts of the pandemic on food and nutrition security. The objectives of this study are to describe demographic characteristics of low-income Vermonters who did and did not participate in federal nutrition assistance programs, and to understand the specific experiences of SNAP, WIC and school meal participants during the early months of the COVID-19 pandemic in Vermont, including relative ease of interacting with the program and perceptions of benefit adequacy to meet household needs during the pandemic. We also examine potential outcomes of program participation, including food security, fruit and vegetable intake, and perceived stress, with a focus on low-income Vermonters who participate in federal nutrition assistance programs. An in-depth understanding of the challenges faced in this novel social environment is needed to guide efforts to adapt nutrition support systems to better meet the needs of vulnerable individuals during this ongoing crisis and future crises. The following paragraphs review the literature relevant to these topics.

## Materials and methods

### Data collection

This study used survey data collected by the National Food Access and COVID Research Team (NFACT), a multistate collaborative effort [68]. Survey questions examined various aspects of Vermonters’ experiences with food access and food security and related worries during the pandemic, in addition to a broad set of demographic characteristics [68]. Multiple iterations of the survey, beginning in March 2020, have been administered, both within the state of Vermont and nationally, with modifications occurring at each stage. This study incorporated data collected online between July 29, 2020 and September 17, 2020 from a sample of Vermont residents recruited via email by the survey research firm Qualtrics. To recruit participants, Qualtrics partners with market research services that maintain pools of respondents who have agreed to be contacted to participate in surveys. The sample of 600 Vermonters (age 18 and older) reflects the state’s population profile with respect to race, ethnicity and income [68]. Representativeness of the sample was achieved through quotas to match characteristics of the state’s race, ethnicity and income distributions. Participants provided informed consent prior to beginning the survey. The survey took approximately 35 min to complete.

### Relevant variables

Independent variables for this study included select demographic characteristics and binary variables reflecting participation in three federal nutrition assistance programs: SNAP, WIC and school meals. Of note, we classified recipients of the special Pandemic Electronic Benefits Transfer (P-EBT) program, offered to families of children who would have received free or reduced school meals prior to shutdowns, as school meal participants. To compensate for relatively small sizes within each category, most demographic variables (e.g., gender, race) were condensed or analyzed as binary (Supplementary Table 1).

Variables were also created to reflect participation in multiple or any federal nutrition assistance program. As one aim of this study was to distinguish between low-income and other program participants, we created a variable to reflect participants that fell above or below 200% of the federal poverty level (FPL) based on household size. Following the approach for the National Center for Children in Poverty, we chose this cutoff point to ensure our low-income group included both the poor and near poor [69]. Further, this threshold is the typical cutoff point for participation in Medicaid in the state of Vermont [70]. To create this variable, first, average household income was calculated based on reported categories. To calculate, we took the midpoint of each income category (i.e., if the respondent reported a household income between $10,000 and $14,999, this was averaged to $12,499.50). These midpoints were compared to 200% of the FPL based on reported household size [71]. If a respondent’s average household income fell below the 200% threshold, they were classified as low income.

We also evaluated four dependent variables based on self-reported data: food security, fruit intake, vegetable intake, and perceived stress (additional details in Supplementary Table1). Food security status was evaluated using the validated USDA 6-item short-form food security module, modified to reflect experiences since the start of the pandemic (March 2020) [72]. Following established scoring procedures, respondents who answered 2 or more out of the 6 survey questions positively were classified as food insecure [72].

Survey respondents reported perceived fruit and vegetable intake based on binned categories (0 = None, 1 = ½ cup or less, 2 = ½ to 1 cup, 3 = 1–2 cups, 4 = 2–3 cups, 5 = 3–4 cups, 6 = 4 cups or more). For analyses, these were condensed to reflect whether perceived intake did or did not meet USDA guidelines for fruit and vegetable intake [73]. Given that established thresholds for fruit (2 cups) and vegetable (2.5 cups) intake did not neatly correspond with survey categories, any respondents reporting fruit intake of “1–2 cups” or more and vegetable intake of “2–3 cups” or more of vegetables were classified as meeting intake recommendations. Accordingly, our recategorization may slightly overestimate the proportion of respondents who meet fruit and vegetable intake recommendations.

Stress was measured using the validated four-item perceived stress scale [74], which poses a series of scenarios to which respondents indicate that they occur never (0) to very often [4]. The scale was corrected for all questions so that higher scores reflect higher stress, which requires reverse scoring on two of the four questions. Results were then summed for an overall perceived stress score of 0–16. To our knowledge, there is no established cut off to establish a “high” score.

Finally, a new set of questions developed by our team asked respondents who participated in federal nutrition assistance programs to respond on a five-point Likert scale (strongly disagree to strongly agree) to several statements regarding their experiences with the programs. Participants were given the option to make further optional comments on their experiences.

### Data analysis

We used descriptive statistics to assess individual demographic characteristics of federal nutrition assistance program participants and their experiences with these programs during the early months of the COVID-19 pandemic, and bivariate tests (chi-squared or t-tests, based on data type) to assess demographic differences between low-income program participants and non-participants with an alpha level of 0.05 indicating significant differences. Where sample size allowed, statistical tests were conducted on SNAP (*n* = 114) and WIC (*n* = 25) participants separately. We also summarized open-ended comments (*n* = 70) provided by nutrition assistance program participants about the programs. Notably, open-ended responses were optional, and many respondents elected not to provide substantive comments, such that broad trends are difficult to identify, particularly among the small WIC subsample. Given the small sample of qualitative data, comments were divided by relevant program and coded into three broad themes using NVIVO version 20 [75]: program challenges, program benefits, or both.

In order to estimate the effects of federal nutrition assistance program participation on food security, fruit and vegetable intake, and perceived stress, we used chi-square tests, t-tests, and nearest neighbors matching techniques. Nearest neighbors matching is useful to approximate treatment effects where only observational data is available [76]. In simple terms, nearest neighbors matching techniques employ a quasi- experimental method to attempt to compensate for selection bias by selecting those untreated individuals who are most similar to a sample of treated individuals based on a set of predefined relevant and observable characteristics. In the context of this study, federal nutrition assistance program participation served as the treatment in various combinations. However, given the significant variation in programming between school meal programs, SNAP and WIC, we ran matching analyses on participants in three ways: (1) participation in any nutrition assistance program; (2) participants in school meal program; and (3) participants in SNAP/WIC. Since school meal eligibility during this time was universal rather than income-based, we explored this relationship separately from SNAP and WIC program participation. We combined SNAP and WIC participants together as a single treatment group due to the small sample size of WIC participants. While we acknowledge that combining SNAP and WIC participants into a single group of participants utilizing a federal program is not ideal, given that WIC participation requires children in the household or pregnancy, we attempted to control for this difference by matching on the presence of children in the household. All analyses were stratified by income (low/high) to assess the differential impacts of nutrition assistance program participation on these groups. In each of these analyses, we matched program participants to non-participants based on a set of six demographic covariates that are likely to be associated with program participation or relevant outcomes: age (under 35), children in household, negative job change, education (at least a bachelor’s degree), household size (4 or more individuals), and rurality. Given the highly correlated nature of income to education and negative job change, and the fact that we further stratified analyses by low/high income thresholds, we did not match on income.

We used a k-nearest neighbor matching approach, which uses the most similar non-treated respondents (k) to compare to each treated federal program participant. Previous research suggests that this k-nearest neighbor matching approach works well with eight or fewer covariates that we utilize [76, 77]. Our analysis used the Mahalanobis distance, which works well in instances of using fewer covariates between the treated/non-treated observations to identify matches [77, 78]. Matches are selected based on the shortest “distance” that can be found, but to ensure quality matches we set a maximum caliper for each analysis at the smallest caliper (shortest distance) that allowed at least 2 matches to be found for all matches. Calipers varied slightly between analyses, but all were set at a maximum of between 0.15 and 0.25, with higher calipers employed for analyses in which matches were more difficult to find. To satisfy the common support condition [76], we reported the minimum (always two) and maximum matches, total number of treated, and matched individuals in all our models.

Using these matches, we reported average treatment effect on the treated (ATET), which assesses the difference between expected outcomes (food security, fruit intake, vegetable intake, and perceived stress) with and without treatment (nutrition assistance programs) for those who participate in treatment [76]. All analyses were conducted using Stata 16 [79].

## Results

### Section 1: descriptive statistics

#### Sample demographics

Table 1 depicts demographic characteristics by group. About one in three respondents (*n* = 202) were classified as low-income. Of the full sample (*N* = 600), 44.2% were over 55 years, but only 28.2% fell into this category when restricted to low-income respondents, with the largest proportion of this group aged 18–34 (40.6%). Most respondents were female in both the full sample (67.3%) and the low-income group (76.2%). Average household size was 2.61 people (std. dev = 1.569) for the full sample and 2.93 people (std. dev = 2.034) for low-income participants, with 29.7% and 41.1% reporting children in the household, respectively. Of total respondents, 8.2% identified as BIPOC and/or Hispanic ethnicity as compared to 9.9% within the low-income category. Within the full sample, 47.7% of respondents had at least a college degree, whereas only 21.3% of low-income fell into this category. 45% of total respondents and 52.5% of low-income respondents lived in households that experienced a negative job change during the first 4–5 months of the pandemic, including job loss, furlough or reduction in hours. Only 35.3% of total respondents and 28.7% of low-income respondents lived in an urban setting.

**Table 1:** Demographic characteristics of full sample, low-income respondents and federal nutrition assistance program participants

Demographic Characteristic			Full Sample (*N* = 600)	All Low Income (*n* = 202)	Low-income non-participants (*n* = 86)	School Meals (*n* = 68)	Any Program (*n* = 164)	SNAP and WIC Only (*n* = 124)
Age Group (%)	18–34 years 35–54 years 55 years+		26.2 (157) 29.7 (178) 44.2 (265)	40.6 (82) 31.2 (63) 28.2 (57)	50.0 (43) 20.9 (18) 29.1 (25)	44.1 (30) 50.0 (34) 5.9 (4)	34.8 (57) 41.5 (68) 23.8 (39)	33.1 (41) 38.7 (48) 28.2 (35)
Gender ID	Female Not Female		67.3 (404) 32.7 (196)	76.2 (154) 23.8 (48)	83.7 (72) 16.3 (14)	70.6 (48) 29.4 (20)	69.5 (114) 30.5 (50)	68.5 (85) 31.5 (39)
Income	Less than $10,000 $10,000 to $24,999 $25,000 to $49,999 $50,000 to $74,999 $75,000 to $99,999 $100,000 or more		6.2 (37) 14.0 (84) 23.8 (143) 17.3 (104) 14.5 (87) 24.2 (145)	17.8 (36) 40.1 (81) 39.1 (79) 2.5 (5) 0.5 (1) 0 (0)	16.3 (14) 33.7 (29) 46.5 (40) 2.3 (2) 1.2 (1) 0.0 (0)	8.8 (6) 17.6 (12) 26.5 (18) 16.2 (11) 22.1 (15) 8.8 (6)	13.4 (22) 32.3 (53) 26.8 (44) 11.0 (18) 10.4 (17) 6.1 (10)	16.1 (20) 38.7 (48) 27.4 (34) 8.9 (11) 4.8 (6) 4.0 (5)
Children	No children in HH Children in HH		70.0 (415) 30.0 (178)	58.9 (119) 41.1 (83)	68.6 (59) 31.4 (27)	11.8 (8) 88.2 (60)	42.9 (70) 57.1 (93)	52.0 (64) 48.0 (59)
Household Size	1–2 members 3 or more members		60.2 (357) 39.8 (236)	46.5 (94) 53.5 (108)	44.2 (38) 55.8 (48)	14.7 (10) 85.3 (58)	41.1 (67) 58.9 (96)	49.6 (61) 50.4 (62)
BIPOC	BIPOC Not BIPOC		8.2 (49) 91.8 (551)	9.9 (20) 90.1 (182)	11.6 (10) 88.4 (76)	8.8 (6) 91.2 (62)	10.4 (17) 89.6 (147)	12.1 (15) 87.9 (109)
Education	High School or less Some college/Associate College degree or more		19.0 (114) 33.3 (200) 47.7 (286)	33.7 (68) 45.0 (91) 21.3 (43)	32.6 (28) 34.9 (30) 32.6 (28)	29.4 (20) 39.7 (27) 30.9 (21)	31.1 (51) 47.0 (77) 22.0 (36)	34.7 (43) 46.8 (58) 18.5 (23)
Job Disruptions	Any job change No job change		46.2 (270) 53.8 (314)	54.4 (106) 45.6 (89)	56.1 (46) 43.9 (36)	57.4 (39) 42.6 (29)	54.0 (87) 46.0 (74)	52.1 (63) 47.9 (58)
Rural/Urban Residence	Urban Rural		35.4 (212) 64.6 (387)	28.9 (58) 71.1 (143)	23.5 (20) 76.5 (65)	27.9 (19) 72.1 (49)	32.9 (54) 67.1 (110)	34.7 (43) 65.3 (81)
Low-income	Low-income Not low-income		33.7 (202) 66.3 (398)	-- --	-- --	50.0 (34) 50.0 (34)	70.1 (115) 29.9 (49)	80.6 (100) 19.4 (24)

*Note*. Sample size is adjusted for several variables based on missing data. Within the full sample, *n* = 593 for children in household and household size; *n* = 594 for job disruptions; *n* = 599 for rural/urban residence. Within the low-income non-participants, *n* = 85 for rural/urban residence

### Federal nutrition assistance program participation

Of all respondents, 27.3% (*n* = 164) reported that their household used at least one federal nutrition assistance program and 5.67% (*n* = 34) reported that their household used two or more programs. Divided by program, 68 respondents participated in a school meal program, 114 participated in SNAP and 25 participated in WIC.

Over half (56.9%, *n* = 115) of low-income respondents participated in at least one federal nutrition assistance program (Table 2). Among these respondents, there was a significant association between age and program participation, with 47.6% of 18–34-year-olds participating, compared to 71% of 35–54-year-olds and 56.1% of those 55 and over. We also found significant associations between program participation and gender (x^2^ [1] = 4.778, *p* = 0.029), and presence of children in the household (x^2^ [1] = 6.075, *p* = 0.014) with higher participation among those who did not identify as female and those living in a household with children. Finally, we found a significant association between program participation and education, with the highest rates of participation among those who had some college or an associate degree (compared to those with more or less education), and the lowest rates of participation among those with a college or advanced degree.

We also found significant associations between program participation and gender (x2 [1] = 4.778, *p* = 0.029), presence of children in the household (x2 [1] = 6.075, *p* = 0.014) and education, with higher participation among those who did not identify as female, had some college or an associate’s degree (compared to those with more or less education), and those living in a household with children.

**Table 2:** Crosstab of federal nutrition assistance program participation by select demographic characteristics among low-income respondents (*n* = 201)

Variable		Participating in any program (*n* = 115), n (%)	Not participating in any program (*n* = 86), n (%)	*P* Value
Age	18–34 years* 35–54 years* 55 years+	39 (47.6) 44 (71.0) 32 (56.1)	43 (52.4) 18 (29.0) 25 (43.9)	0.019
Gender Identity	Female Not Female	81 (52.9) 34 (70.8)	72 (47.1) 14 (29.2)	0.029
Children	No children in HH Children in HH	59 (50.0) 56 (67.5)	59 (50.0) 27 (32.5)	0.014
Household Size	1–2 members 3 or more members	55 (59.1) 60 (55.6)	38 (40.9) 48 (44.4)	0.609
BIPOC	BIPOC Not BIPOC	10 (50.0) 105 (58.0)	10 (50.0) 76 (42.0)	0.492
Education	High School or less Some College/Associate* College or advanced degree*	40 (58.8) 60 (66.7) 15 (34.9)	28 (41.2) 30 (33.3) 28 (65.1)	0.002
Job Disruptions	Any job change No job change	60 (56.6) 52 (59.1)	46 (43.4) 36 (40.9)	0.727
Rural/Urban Residence	Urban Rural	37 (64.9) 78 (54.5)	20 (35.1) 65 (45.5)	0.181

*Note*. Sample size is adjusted for several variables based on missing data. One low-income was excluded for missing data on program participation. For non-program participants, *n* = 83 for job disruptions; *n* = 85 for rural/urban residence. For program participants, *n* = 112 for job disruptions

*Categories significantly different

### Program experiences

**Fig. 1:**
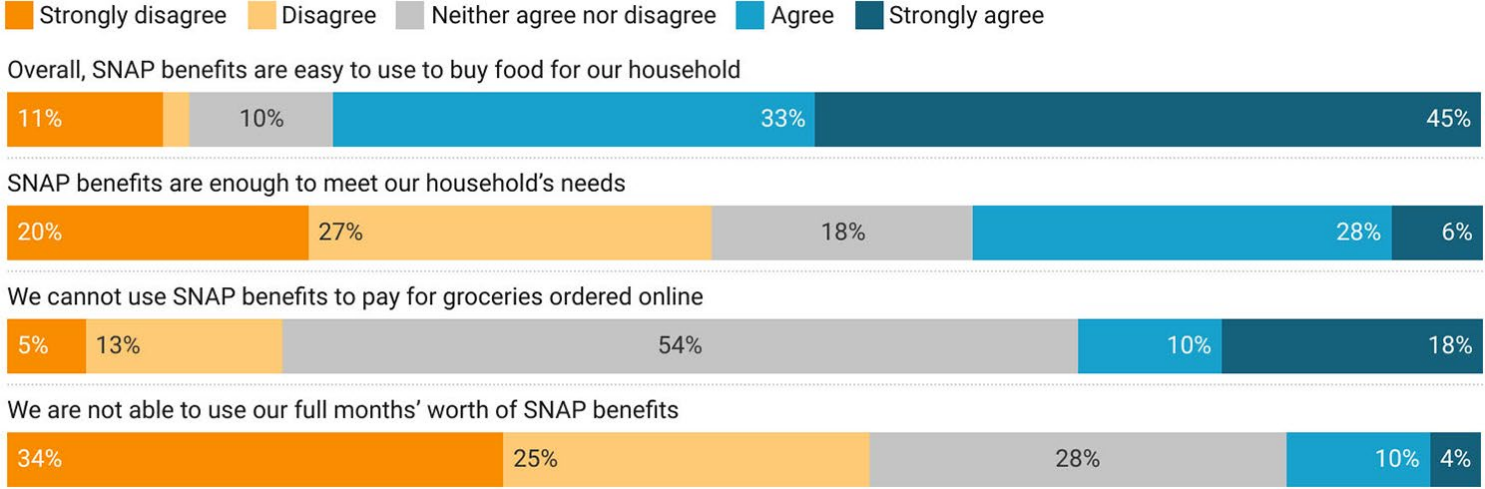
Experiences of SNAP participants during the early months of the COVID-19 pandemic in Vermont

**Fig. 2:**
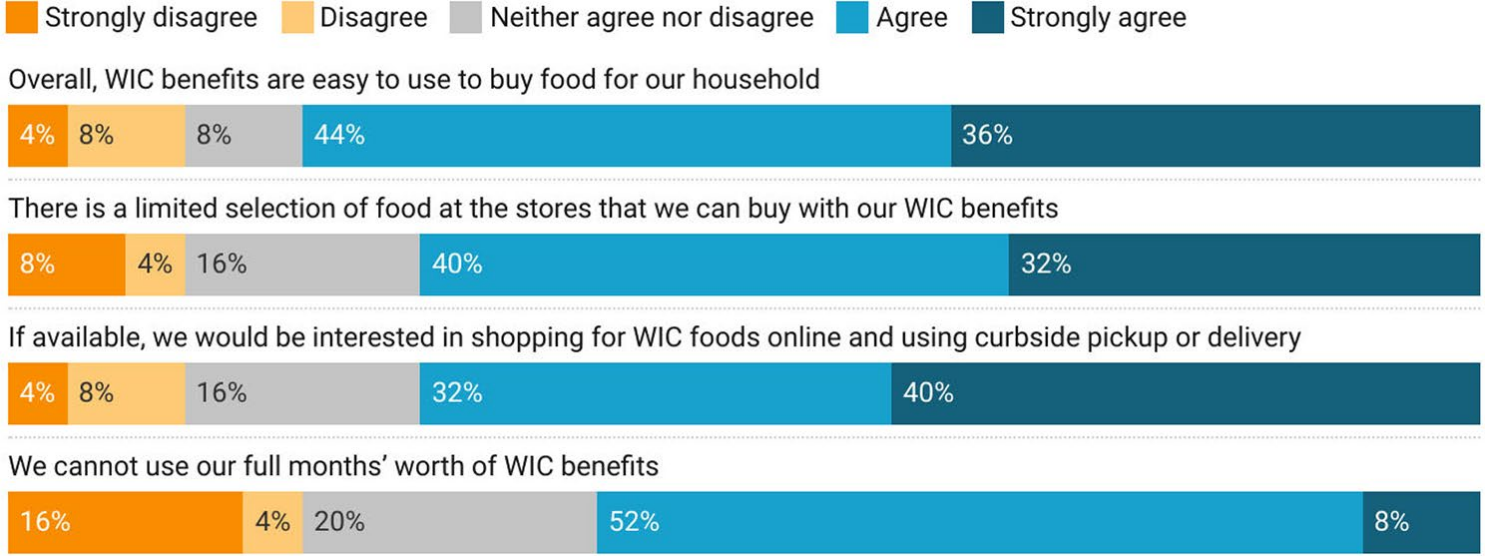
Experiences of WIC participants during the early months of the COVID-19 pandemic in Vermont

When asked to express their level of agreement with a series of position statements, most of both SNAP and WIC participants, 78% and 80% respectively, agreed or strongly agreed that the benefits are easy to use (Figs. 1 and 2). Only 14% of SNAP participants agreed with the statement “we are not able to use our full months’ worth of SNAP benefits,” while, 60% of WIC participants agreed or strongly agreed that they could not use a full month’s worth of WIC benefits. However, nearly half (47%) of participants disagreed with the statement “SNAP benefits are enough to meet our household’s needs,” suggesting that benefits alone did not fully compensate for household food security needs. Just over half (54%) of SNAP participants neither agreed nor disagreed that they were unable to use their benefits to order groceries online, which was available from some retailers during the pandemic, suggesting that these respondents may not have attempted to do so. However, of WIC participants, 72% agreed or strongly agreed that they would be interested in online shopping for WIC foods with delivery or curbside pickup options. 72% also agreed with the observation that there is a limited selection of foods that can be purchased with WIC benefits.

**Fig. 3:**
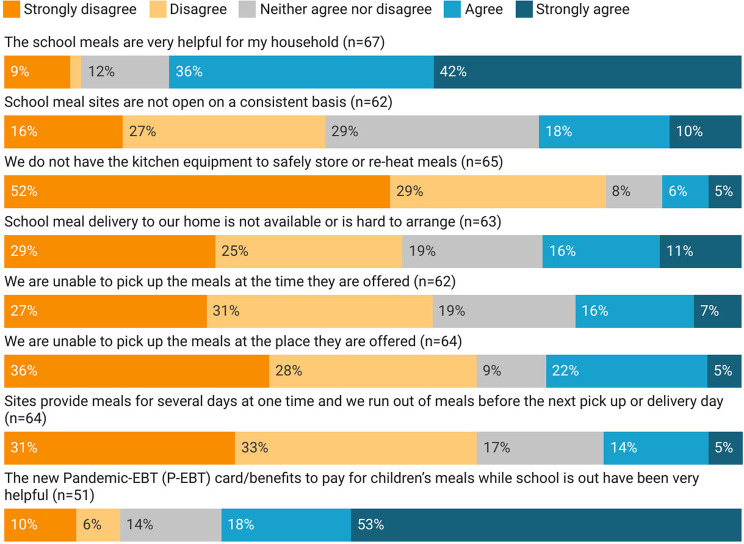
Experiences of School Meals recipients during the early months of the COVID-19 pandemic in Vermont

**Fig. 4:**
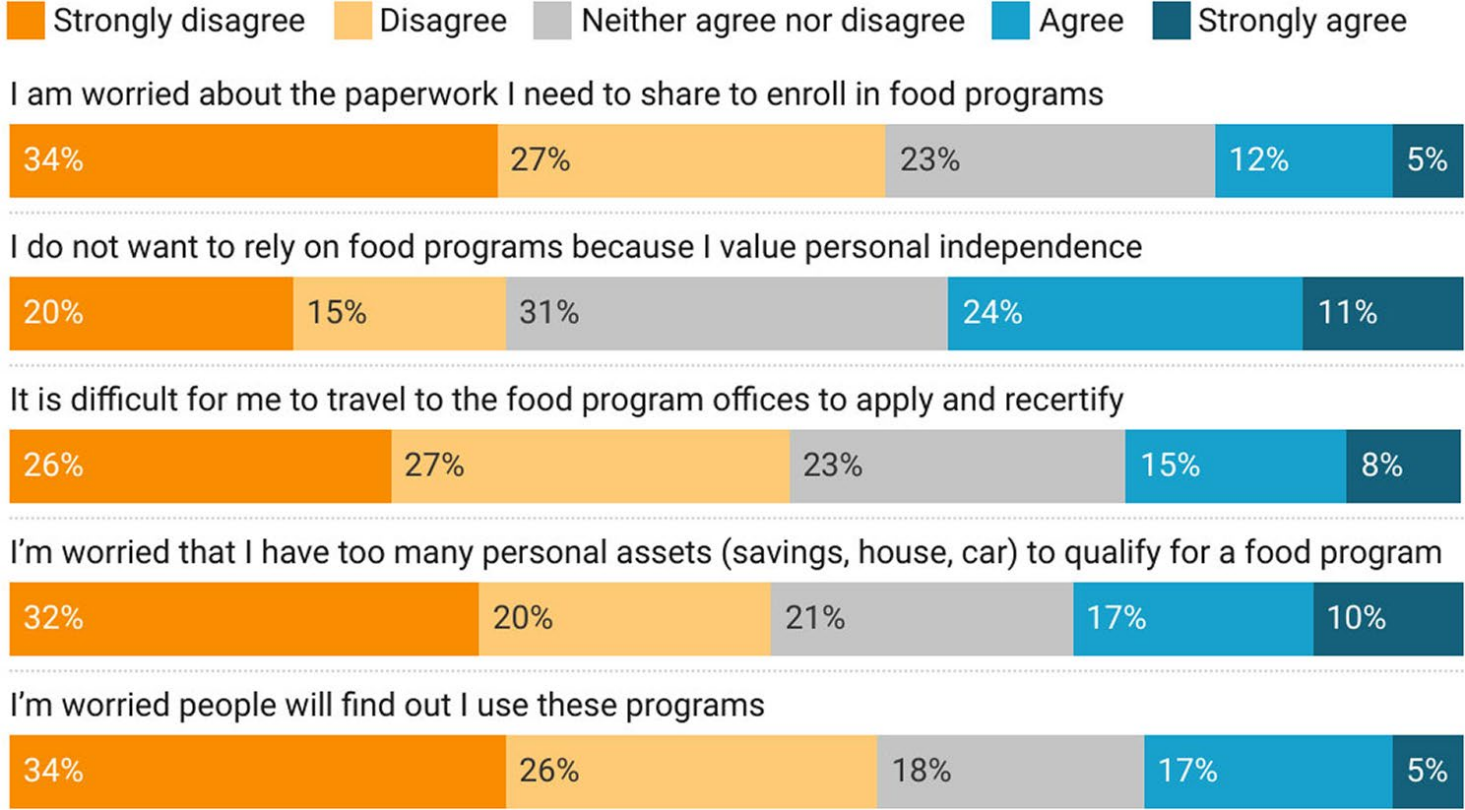
Experiences of federal nutrition assistance program participants during the COVID-19 pandemic in Vermont

Most school meal participants (78%) and P-EBT recipients (71%) agreed that these programs had been helpful to their families (Figs. 3 and 4). When asked to report their level of agreement with specific challenges related to school meals during the pandemic, the most common complaints were that school meal sites were not consistently open (28%), home delivery was not available or was difficult (27%), and that participants were unable to pick up at the sites (27%) and times (23%) offered. Fewer than 20% of participants reported running out of meal provisions before the next delivery dates (19%) or limitations related to inadequate kitchen equipment needed to store and reheat meals (11%).

Among all program participants, 35% agreed that they did not want to rely on nutrition assistance programs because they valued personal independence and 22% expressed worry that others would find out they used programs. Others expressed pragmatic concerns with qualifying and recertifying for programs, including possessing too many personal assets to be eligible (27%), difficulties travelling to program sites for appointments (23%), and worries over the paperwork needed to enroll (17%).

In open-ended comments, participants responded with a mix of gratitude for the programs along with discussion of challenges and limitations. About a quarter of WIC participants provided further comments on their experiences, of which a couple commented that the selection of foods offered was limited, and not always available during the pandemic, e.g., “its been harder to get certain WIC items since COVID”. Over a dozen SNAP participants, or roughly a third of those who provided qualitative data, commented on the helpfulness of the program during the pandemic, with particular emphasis on the necessity of the temporary increase in benefits provided: “the increase was very much appreciated and needed”; “the extra money is necessary for both before and after the pandemic”.

However, echoing responses to closed-ended questions, numerous SNAP participants elaborated on challenges they faced with their benefits. Some argued that, even with temporary increases, benefits were inadequate to meet their needs, whether due to rising costs or supply shortages: “It’s not enough given the rising costs of everything,” said one participant, while another stated, “I feel like the benefits didn’t go as far because I had to buy name brand items due to [the] store brand [being] sold out”. Another respondent observed cyclical challenges associated with benefits noting that “everyone shops on the first of the month, if the store is out, some people go without. I get SNAP & SSI [social security insurance], and my money is all gone by the 10th of every month”. Other observed challenges included bureaucratic issues in qualifying, limited benefit eligibility due to age and seemingly arbitrary changes to benefits, as well as limited opportunities to shop online.

Participants were also given the opportunity to comment on the P-EBT and school meal programs, resulting in substantive comments from about 20 participants. Most responses suggested that the programs had been helpful: “I don’t know what we would have done with[out] the school meals. We appreciate them more than many people can imagine.” However, a small subset reiterated that the programs were still “Not enough to feed the kids,” or wished for their continuation, i.e., “P-EBT was a blessing and I wish we had more”. Although few specific challenges were discussed, one respondent did note that their family did not prefer the taste of school meals, and another that delivery options were important to the value of school meals during the COVID-19 pandemic: “When meals were being delivered by bus, they were very helpful. Grocery stores didn’t have items we needed in stock, and buying groceries was more expensive than school lunches had been. Getting food deliveries was very helpful to my family. When they stopped delivering, we were unable to pick up meals at the allotted time.”

### Outcome variables

Among all respondents, 71.0% were consistently food secure since the start of the COVID-19 pandemic (Table 3). Most did not meet USDA recommendations for either fruit intake (58.5%) or vegetable intake (72.3%). The average perceived stress score (out of 16) was calculated to be 6.85 for the full sample. Low-income respondents were significantly less likely than higher income respondents to meet fruit and vegetable recommendations (*p* < 0.001) and were significantly more likely to have experienced food insecurity and higher perceived stress since the start of the COVID-19 pandemic (*p* < 0.001).

**Table 3:** Dependent variable frequencies for full sample, low-income respondents, and federal nutrition program participants

Outcome Variables		Full Sample (*N* = 600)	All Low-Income (*n* = 202)	Low-Income No Programs (*n* = 86)	School Meals (*n* = 68)	Any Program (*n* = 164)	SNAP and WIC (*n* = 124)
Fruit Recommendation	Met Did not meet	41.5 (249) 58.5 (351)	29.2 (59) 70.8 (143)	36.0 (31) 64.0 (55)	39.7 (27) 60.3 (41)	32.9 (54) 67.1 (110)	29.0 (36) 71.0 (88)
Vegetable Recommendation	Met Did not meet	27.7 (166) 72.3 (434)	15.3 (31) 84.7 (171)	26.7 (23) 73.3 (63)	17.6 (12) 82.4 (56)	14.6 (24) 85.4 (140)	12.1 (15) 87.9 (109)
Food Security	Food Secure Food Insecure	71.0 (414) 29.0 (169)	39.9 (77) 60.1 (116)	50.6 (41) 49.4 (40)	53.8 (35) 46.2 (30)	42.5 (68) 57.5 (92)	36.1 (44) 63.9 (78)
Perceived Stress Score		6.85	8.37	8.49	7.35	7.98	8.17

*Note*. Sample size is adjusted for several variables based on missing data. For the food security variable, *n* = 583 for the full sample; *n* = 193 for the low-income sub-sample; *n* = 81 for program non-participants; *n* = 65 for school meal participants; *n* = 160 for all program participants; *n* = 122 for SNAP/WIC participants. For the perceived stress variable, *n* = 597 for the full sample; *n* = 201 for the low-income subsample; *n* = 163 for all program participants; *n* = 123 for SNAP/WIC participants

### Nutrition assistance program participation and outcomes

#### Federal nutrition assistance program participation and food security

Among low-income participants, food insecurity was associated with SNAP/WIC participation (*p* = 0.031), but not school meal participation through chi-square tests (*p* = 0.031).

Using matching techniques to approximate the effects of federal nutrition assistance program participation on food security, we found a significant association between participation in any program and increased food insecurity since the start of the COVID-19 pandemic, both for the higher income (*p* = 0.001) and the low-income group (*p* < 0.001) (Table 4). In other words, among similar higher-income households, those using any federal nutrition assistance program were more likely to be food insecure compared to those who are not using any program. The same was found when comparing among otherwise similar low-income households. When school meal participation and SNAP/WIC participation were evaluated as separate treatments, we found that this association held true for SNAP/WIC participation among respondents that were not low-income (*p* < 0.001).

**Table 4:** Food insecurity of federal nutrition assistance program participants as compared to non-participants using matching analysis

		Coefficient	Robust Std. Error	*p*=	95% CI		Minimum matches	Maximum matches	Raw Control/Treated *n*	Treated matched	Total *n* matched
Higher Income Respondents	SNAP/WIC	0.313	0.030	< 0.001*	0.254	0.373	2	28	433/117	117/117	234
	School Meals	0.086	0.098	0.383	-0.107	0.279	2	4	367/181	64/64	134
	Any Program	0.256	0.077	0.001*	0.105	0.407	2	27	483/64	181/181	362
Lower Income Respondents	SNAP/WIC	0.087	0.087	0.317	-0.083	0.256	2	10	433/117	117/117	234
	School Meals	-0.107	0.105	0.305	-0.313	0.097	2	8	483/64	64/64	134
	Any Program	0.323	0.089	< 0.001*	0.149	0.498	2	12	367/181	181/181	362

*Note*. Each program participation variable was used as a “treatment” in a separate matching analysis while using six demographic controls (gender, children in household, education negative job change, household size, rural/urban) to conduct the matching. Negative coefficients reflect an association with increased food security

### Federal nutrition assistance program participation and fruit and vegetable intake

We used matching analysis and chi-square tests to evaluate associations between program participation and fruit and vegetable intake. Using chi-square tests we found a significant association between SNAP/WIC program participation and reduced probability of meeting fruit intake recommendations in both the full sample (*p* = 0.001) and low-income subgroup (*p* = 0.049), but not for school meals. This trend was only significant in the full sample when all programs were grouped (*p* = 0.008). When SNAP and WIC were examined separately, only SNAP participation within the full sample was significantly associated with reduced fruit intake (*p* = 0.001). When matching analysis was used to account for select demographic controls, we found no significant associations for individual programs, but we did see a significant association between participation in any program and reduced probability of meeting fruit intake recommendations within the low-income group (Table 5; *p* = 0.048). Meaning that for low-income households participating in a program, compared to other low-income households not participating in a program, there was lower likelihood of meeting fruit intake recommendations.

**Table 5:** Fruit intake of federal nutrition assistance program participants as compared to non-participants using nearest neighbors matching analysis

		Coefficient	Robust Std. Error	*p*=	95% CI		Minimum matches	Maximum matches	Raw Control/Treated *n*	Treated matched	Total *n* matched
Higher Income Respondents	SNAP/WIC	0.006	0.009	0.483	-0.012	0.025	2	28	447/119	119/119	238
	School Meals	0.002	0.116	0.986	-0.225	0.229	2	5	495/65	67/67	134
	Any Program	0.048	0.088	0.590	-0.125	0.220	2	27	377/186	186/186	372
Lower Income Respondents	SNAP/WIC	-0.067	0.082	0.414	-0.227	0.094	2	10	447/119	119/119	238
	School Meals	-0.097	0.114	0.393	-0.320	0.126	2	7	495/67	67/67	134
	Any Program	-0.183	0.092	0.048*	-0.363	-0.002	2	12	377/186	186/186	372

*Note*. Each program participation variable was used as a “treatment” in a separate matching analysis while using six demographic controls (gender, children in household, education negative job change, household size, rural/urban) to conduct the matching. Negative coefficients reflect an association with a reduced probability of meeting recommended fruit intake levels

Using chi-square tests we found a significantly reduced probability of meeting vegetable recommendations for both SNAP/WIC participants (*p* < 0.001) and all participants grouped (*p* < 0.001). Within the full sample, this trend held for SNAP but not WIC when each was examined alone, but sample size precluded conducting this same analysis within the low-income subsample. We found a weaker association between SNAP/WIC participation and a reduced probability of meeting vegetable recommendations within the low-income group by matching analysis (Table 6; *p* = 0.035).

**Table 6:** Vegetable intake of federal nutrition assistance program participants as compared to non-participants using nearest neighbors matching analysis

		Coefficient	Robust Std. Error	*p*=	95% CI		Minimum matches	Maximum matches	Raw Control/Treated *n*	Treated matched	Total *n* matched
Higher Income Respondents	SNAP/WIC	0.050	0.117	0.669	-0.179	0.279	2	28	447/119	119/119	238
	School Meals	-0.106	0.108	0.328	-0.317	0.106	2	5	495/67	67/67	134
	Any Program	0.075	0.079	0.346	-0.081	0.230	2	27	377/186	186/186	372
Lower Income Respondents	SNAP/WIC	-0.122	0.580	0.035*	-0.235	-0.009	2	10	447/119	119/119	238
	School Meals	-0.111	0.075	0.141	-0.259	0.037	2	7	495/67	67/67	134
	Any Program	-0.101	0.069	0.141	-0.237	0.034	2	12	377/186	186/186	372

*Note*. Each program participation variable was used as a “treatment” in a separate matching analysis while using six demographic controls (gender, children in household, education negative job change, household size, rural/urban) to conduct the matching. Negative coefficients reflect an association with a reduced probability of meeting recommended vegetable intake levels

### Federal nutrition assistance program participation and perceived stress

Within the full sample, we found significantly higher rates of perceived stress among all grouped program participants (*p* < 0.001), SNAP and WIC together (*p* < 0.001) and SNAP participants alone (*p* < 0.001) by t-tests, with an average score of 8.17 for SNAP participants as compared to 6.53 for non-participants. However, school meal and WIC participants analyzed alone did not exhibit significantly higher rates of perceived stress, and no significant associations held for any program when the sample was restricted to only low-income respondents.

We also used matching techniques to examine the effects of program participation on perceived stress (Table 7). The only model for which we found significant effects of program participation on stress scores was that for school meal participation among low-income respondents, wherein we found a significant negative association indicating reduced stress.

**Table 7:** Perceived stress score of federal nutrition assistance program participants as compared to non-participants using nearest neighbors matching analysis

		Coefficient	Robust Std. Error	*p*=	95% CI		Minimum matches	Maximum matches	Raw Control/Treated *n*	Treated matched	Total *n* matched
Higher Income Respondents	SNAP/WIC	0.403	0.535	0.451	-0.645	1.451	2	28	446/118	118/118	236
	School Meals	-0.088	0.779	0.910	-1.615	1.438	2	5	493/67	67/67	134
	Any Program	0.853	0.610	0.162	-0.344	2.059	2	27	376/185	185/185	370
Lower Income Respondents	SNAP/WIC	0.269	0.525	0.608	-0.759	1.297	2	10	446/118	118/118	236
	School Meals	-1.492	0.721	0.039*	-2.906	-0.079	2	7	493/67	67/67	134
	Any Program	1.001	0.575	0.080	-0.121	2.135	2	12	376/185	185/185	370

*Note*. Each program participation variable was used as a “treatment” in a separate matching analysis while using six demographic controls (gender, children in household, education negative job change, household size, rural/urban) to conduct the matching. Negative coefficients reflect an association with a reduced perceived stress score

## Discussion

This study builds on prior literature examining food insecurity during the ongoing COVID-19 pandemic by exploring the role of federal nutrition assistance programs within the context of Vermont, a mostly rural state with a relatively strng policy and social response to the pandemic [65]. We found that, despite shortcomings, participants generally perceived federal nutrition assistance programs as helpful or easy to use. We documented notable levels of food insecurity, suboptimal fruit and vegetable intake, and perceived stress among participants and non-participants alike. Understanding food and nutrition security and perceived stress outcomes under these conditions can provide insights regarding how nutrition assistance programs can provide for the most vulnerable even in such challenging times.

Our findings regarding nutrition assistance program perception correspond closely to prior studies on several counts. In line with calls for increased total SNAP benefit allotment in other studies [8, 80], we found that 47% of participants felt that benefits were not adequate to meet their household’s needs. Likewise, restricted product options available through WIC are a continuing topic of debate [17], which is reflected in our results, although our respondents were not prompted make a value judgment on these limitations. Our results also suggest that online utilization options for SNAP and WIC participants could improve accessibility and efficacy of these programs, which echoes perceptions of convenience and time savings associated with online options in earlier studies [14, 19, 81, 82]. However, in considering expanding online options for these programs, it is important to revisit reported concerns, including associated transaction costs and limited control over selection and quality [81, 83, 84]. Logistical challenges associated with school meal delivery in other studies, such as scheduling conflicts with school breakfasts [26, 27], are more difficult to evaluate against our results, given the altered format of meal delivery during the pandemic. However, new and additional logistical challenges, including accessibility of school meals related to site location, timing, and lack of delivery options continued to pose challenges for a substantial number of participants. At-home options for school meals may overcome other perceived challenges, such as infringement on family mealtimes [27].

Despite a perception of utility among participants, we found that – aligning with prior literature [6, 30] – federal nutrition assistance program participation was significantly associated with food insecurity for both low-income and not low-income respondents, even when we explored this using matching techniques to compare similar households. We also found a reduced probability of meeting fruit intake recommendations for low-income program participants, and a reduced probability of meeting vegetable intake recommendations for low-income SNAP/WIC participants. There is a substantial body of literature supporting the efficacy of federal nutrition assistance programs in alleviating food insecurity and, to some degree, improving diet quality [33, 34, 61]. Our findings should be considered within the unique context of the time and place at which our data were collected, and of course, as subject to limitations in the analytical models employed.

It has been documented that the pandemic exacerbated food insecurity [85] and changed eating patterns. While some studies have found evidence of increased fruit and vegetable intake during the pandemic [86], these impacts were not universal. In a survey of Michigan adults, Litton and Beavers [87] found that food insecure respondents not only consumed fewer fruits and vegetables than their food secure counterparts but were more likely to report decreasing fruit and vegetable consumption in the early months of the pandemic, for reasons including quality, availability, price, desire to reduce store trips, and fears of contamination. While fresh fruit and vegetable prices increased relatively little in comparison with meat, fish and dairy products during the early months of the pandemic [88], challenges in access related to fears of contamination and exposure and increased concerns over spoilage as a consequence of reduced grocery trips [87] should not be discounted. Limitations to online ordering options for SNAP and WIC participants are of particular relevance. While Vermont farmers markets were broadly able to remain open during much of the pandemic, even brief closures posed substantial issues for farmers and patrons alike, and reduced vendor space and preordering requirements upon reopening may have impacted Vermonters who regularly relied on such avenues [89].

Pandemic-specific challenges to food access may be particularly potent in a relatively rural context. Rural populations experience food insecurity at elevated rates, which may be exacerbated by structural barriers affecting access, such as large distances to supermarkets [90, 91]. It is possible that, under these conditions, online delivery or curbside pickup options were more challenging or prohibitively expensive for some rural residents. Additionally, social support systems and community networks may play a unique role in the mitigation of food insecurity in rural settings [92], and constraints associated with new social distancing regulation compliance may have impacted such avenues. Additionally, although roughly one-third of our sample was classified as rural using the Rural Urban Commuting Area 4 category (RUCA) designation, the population density is low across the state, with only approximately 45,000 people, or about 10% of the state’s population residing in its largest city [93]. In combination, exacerbated experiences of food and nutrition insecurity in concert with new structural barriers to nutrition assistance program utilization may have limited the capacity of programs to operate optimally or fully compensate for negative shifts in these outcomes for Vermont’s rural population. These limitations leave room for new and expanded strategies to improve access to healthy foods among program participants.

Although few studies have focused specifically on perceived stress during the pandemic, food insecurity is positively associated with various indicators of poor mental health [57–59]. Some research indicates that this relationship may be attenuated by participation in SNAP and WIC [61, 62], but – in line with our findings – other studies have not identified a moderating effect [64]. Interestingly, we did find that perceived stress was significantly lower among low-income respondents with a household member enrolled in a school food program. Few studies have explicitly evaluated the relationship between school meal participation and perceived stress of household members. Our results merit further investigation – Could school meal delivery or pickup have reduced the necessity of public ventures and associated risk of exposure for some families? Could school meal delivery and pick up options during the pandemic have influenced the frequency of family meals or reduced the burden of meal planning? While Nagata et al. [60] did not examine school meal participation during the pandemic, they did report a mediating effect of free groceries and meals on the association between food insecurity and poor mental health. Similarly, in a qualitative analysis, commercial family meal kit use has been associated with perceived benefits including reduced mental load for family meal providers [94]. Additionally, evidence suggests that frequent family meals may be associated with lower rates of depressive symptoms and stress among parents [95]. While these results cannot be directly applied to the role of school meals during the pandemic, further research could illuminate the pathways through which pandemic school meal formats (including more flexible distribution models) might have impacted perceived stress in the household. It is also relevant to consider that the burden of applying for school meal programs was removed due to the universal delivery approach taken during the time under study.

As with any study, our survey and methods were subject to reasonable limitations. Although our sample size was relatively modest, it had a margin of error of 4% and was intentionally designed to reflect Vermont’s population on key demographic factors including income, race and ethnicity. Due to the limited sample of WIC participants, this population was only analyzed in conjunction with SNAP or both SNAP and school food participants, limiting the capacity of this study to distinguish between the impacts of these distinct programs. By design, our survey captures a breadth of data related to food insecurity during the COVID-19 pandemic but covering a substantial range of material naturally limits the depth of data that can practically be captured in any one area. Additionally, given the evolving nature of the pandemic context, subjects of importance to individual experiences of food and nutrition security continue to shift and emerge over time. For example, the administration of school meal programs was evolving at the time of data collection, making it challenging to clearly assess which specific program components and iterations participants responded to. Additionally, the limited number of open-ended responses collected suggests that the full breadth of participant experiences may not have been captured, although the supplementary qualitative data nonetheless adds depth.

Nearest neighbors matching analysis seeks to address weaknesses associated with selection bias in non-experimental study designs, but in the absence of perfect knowledge, such tools cannot perform perfectly. Although our survey captured many demographic variables known to be associated with food and nutrition security, perceived stress and federal nutrition assistance program participation, these are complex constructs influenced by a myriad of interrelated factors. It is likely that additional confounding variables exist that we were unable to fully evaluate. For example, our matching analysis was not able to account for differences in social support, which has been shown to influence food insecurity and perceived stress. Additionally, we were unable to control for the role that disability, physical health and comprehensive mental or emotional health may have on these outcomes, although such factors are known to interact with the experience of food insecurity [57, 96]. Furthermore, our survey design could not meaningfully capture the specific food environments of participants, which can significantly impact food and nutrition security [90]. Integrating these variables into future analyses might better isolate the impacts of federal nutrition assistance programs on food and nutrition security and perceived stress.

Of note, our matching analysis used binary outcomes to maximize power, except for perceived stress. However, by examining outcomes as binary, our models do not account for changes in the intensity of an outcome. While federal nutrition assistance program participation did not reduce food insecurity in our analysis, it is possible that participants may have experienced a reduction in the degree of food insecurity experienced, which would not be reflected in our models.

## Conclusions

Within the context of a pandemic in the state of Vermont, federal nutrition assistance programs were broadly not adequate to address the experience of food insecurity and stress or increase fruit and vegetable intake. However, participants nonetheless perceived these programs as helpful and may have experienced other benefits, including reduced stress among low-income school meal program participants. Continuing research on the delivery and impacts of nutrition assistance programs, particularly in other rural contexts, is needed to inform their implementation. If federal nutrition assistance programs are to function effectively as vital safety nets, they must continue to evolve as new challenges to food and nutrition security emerge.

### Electronic supplementary material

Below is the link to the electronic supplementary material.

Supplementary Material 1

## Data Availability

The datasets used and/or analyzed during the current study are available from the corresponding author on reasonable request.

## Abbreviations

SNAP: Supplemental Nutrition Assistance Program
WIC: Special Supplemental Nutrition Program for Women, Infants, and Children
USDA: United States Department of Agriculture
HEI: Healthy Eating Index
NHANES: National Health and Nutrition Examination Survey
(P)EBT: (Pandemic) Electronic Benefits Transfer
NFACT: National Food Access and COVID Research Team
FPL: Federal Poverty Level
BIPOC: Black, indigenous and people of color
SSI: Social Security insurance
