# Supplementary material for: Experiences participating in federal nutrition assistance programs during the early months of the COVID-19 pandemic: an investigation in Vermont
Source: Nutr J. 2024 Jul 15;23:74. doi: 10.1186/s12937-024-00963-z (PMC11247766; doi:10.1186/s12937-024-00963-z)
Supplement: Supplementary file 1 — Supplementary Material 1 [file 12937_2024_963_MOESM1_ESM.docx]

Supplementary Table 1. Complete list of variables, questions and scales used in analysis.

| Variable | Survey Question | Scale | |
| --- | --- | --- | --- |
| *Demographic Variables* | | |  |
| Age Group |  | 1= 18-34 years, 2 = 35-54 years, 3 = 55 year+ | |
| Female | Which of the following best describes your gender identity? | 1= Female, 0 = Not Female* | |
| Income | Which of the following best describes your household income range in 2019 before taxes? | 1 = Less than $10,000, 2 = $10,000 to $24,999, 3 = $25,000 to $49,999, 4 = $50,000 to $74,999, 5 = $75,000 to $99,999, 6 = $100,000 or more* | |
| Children | Are there children in your household? | 0 = No children in HH  1 = Yes, children in HH | |
| Household Size |  | 0= 1-2 members; 1=  3 or more members | |
| BIPOC |  | 1 = BIPOC, 0 = Not BIPOC* | |
| Education | What is the highest level of formal education that you have completed? | 1 = High School or less  2 = Some college or Associate degree  3 = Bachelors or Advanced degree* | |
| Job Disruptions | Have you or anyone in your household experienced a loss of income or job since the COVID-19 outbreak (March 11^th^, 2020)? | 1 = Yes, 0 = No* | |
| Rural/Urban Residence | Zip codes that intersect with a Census Urbanized Area or Urban Cluster (RUCA 2.0) | 1 = Urban, 2 = Large Rural, 3 = Small Rural, 4 = Isolated | |
| Low-income | Income less than 200% of the federal poverty level based on household size. | 1 = Low-income, 0 = Not low-income | |
| *Diet, Food Security and Stress Variables* | | |  |
| Fruit Consumption | Determined based on responses to reported fruit intake as compared to USDA recommendation for fruit intake (2 or more cups daily to meet recommendation). Original responses include: 0 = None, 1 = ½ cup or less, 2 = ½ to 1 cup, 3 = 1-2 cups, 4 = 2-3 cups, 5 = 3-4 cups, 6 = 4 cups or more | 1 = Met Recommendation, 0 = Did Not Meet Recommendation | |
| Vegetable Consumption | Determined based on responses to reported vegetable intake as compared to USDA recommendation for vegetable intake (2.5 or more cups daily to meet recommendation). Original responses include: 0 = None, 1 = ½ cup or less, 2 = ½ to 1 cup, 3 = 1-2 cups, 4 = 2-3 cups, 5 = 3-4 cups, 6 = 4 cups or more | 1 = Met Recommendation, 0 = Did Not Meet Recommendation | |
| Food Security | Determined based on the responses to the US Household Food Security Survey Module Six-Item Short Form. | 1 = Food Insecure, 0 = Food Secure | |
| Perceived Stress Scale | Perceived Stress Scale Score calculated based on responses to:  In the last month, how often have you felt that you were unable to control the important things  in your life?  In the last month, how often have you felt confident about your ability to handle your personal  problems?  In the last month, how often have you felt that things were going your way?  In the last month, how often have you felt difficulties were piling up so high that you could not  overcome them? | 0-16 (higher scores reflect higher stress) | |
| *Federal Nutrition Assistance Program Participation Variables* | | |  |
| SNAP Participation | Has your household used SNAP benefits since the start of the outbreak? | 1 = Yes, 0 = No | |
| WIC Participation | Has your household used WIC benefits since the start of the outbreak? | 1 = Yes, 0 = No | |
| School Meals Participation | Has your household used a school meal program since the start of the outbreak? | 1 = Yes, 0 = No | |
| *Program Experience Variables* | | |  |
| SNAP Questions | SNAP benefits are enough to meet our household’s needs  Overall, SNAP benefits are easy to use to buy food for our household | 1 = Strongly disagree, 2 = Disagree, 3 = Neither agree nor disagree, 4 = Agree, 5 = Strongly agree | |
|  | We cannot use SNAP benefits to pay for groceries ordered online  We are not able to use our full months’ worth of SNAP benefits | 1 = Strongly agree, 2 = Agree, 3 = Neither agree nor disagree, 4 = Disagree, 5 = Strongly disagree | |
| Additional SNAP Comments |  | Open-ended | |
| WIC Questions | Overall, WIC benefits are easy to use to buy food for our household  There is a limited selection of food at the stores that we can buy with our WIC benefits  We cannot use our full months’ worth of WIC benefits (because, for example, it is hard to go shopping or stores are sold out of WIC items)  If available, we would be interested in shopping for WIC foods online and using curbside pickup or delivery | 1 = Strongly disagree, 2 = Disagree, 3 = Neither agree nor disagree, 4 = Agree, 5 = Strongly agree | |
| Additional WIC Comments |  | Open-ended | |
| School Meals Questions | The school meals are very helpful for my household  School meal sites are not open on a consistent basis  We do not have the kitchen equipment to safely store or re-heat meals  School meal delivery to our home is not available or is hard to arrange  We are unable to pick up the meals at the time they are offered  We are unable to pick up the meals at the place they are offered  Sites provide meals for several days at one time and we run out of meals before the next pick up or delivery day  The new Pandemic-EBT (P-EBT) card/benefits to pay for children’s meals while school is out have been very helpful | 1 = Strongly disagree, 2 = Disagree, 3 = Neither agree nor disagree, 4 = Agree, 5 = Strongly agree, 99 = I don't know | |
| Additional School Meals Comments |  | Open-ended | |
| Questions for All Program Participants | I am worried about the paperwork I need to share to enroll in food programs  I do not want to rely on food programs because I value personal independence  It is difficult for me to travel to the food program offices to apply and recertify  I’m worried that I have too many personal assets (savings, house, car) to qualify for a food program  I’m worried people will find out I use these programs | 1 = Strongly disagree, 2 = Disagree, 3 = Neither agree nor disagree, 4 = Agree, 5 = Strongly agree, 99 = I don't know | |

Note. For these variables, the start of the outbreak is defined as March 11, 2020.

* Original categories were condensed due to small sample sizes.
